# Field-testing of the rapid assessment of disability questionnaire

**DOI:** 10.1186/1471-2458-14-900

**Published:** 2014-09-01

**Authors:** Manjula Marella, Lucy Busija, Fakir M Amirul Islam, Alexandra Devine, Kathy Fotis, Sally M Baker, Beth Sprunt, Tanya J Edmonds, Nafisa Lira Huq, Anaseini Cama, Jill E Keeffe

**Affiliations:** Nossal Institute for Global Health, University of Melbourne, Level 4, Alan Gilbert Building, 161 Barry Street, Carlton, VIC 3010 Australia; Centre for Eye Research Australia, Royal Victorian Eye and Ear Hospital, University of Melbourne, Melbourne, Victoria Australia; Deakin Biostatistics Unit, Faculty of Health, Deakin University, Melbourne, Victoria Australia; Department of Statistics, Data Science and Epidemiology, Faculty of Health, Arts and Design, Swinburne University of Technology, Melbourne, Victoria Australia; Centre for Reproductive Health, International Centre for Diarrheal Disease Research, Dhaka, Bangladesh; CMW Hospital Eye Department, Ministry of Health, Suva, Fiji; International Agency for the Prevention of Blindness, Western Pacific Region, Pacific Secretariat, Suva, Fiji; L V Prasad Eye Institute, Hyderabad, India

**Keywords:** Disability, Rapid assessment, Validation, Questionnaire, Inclusive development

## Abstract

**Background:**

The Rapid Assessment of Disability (RAD) questionnaire measures the magnitude and impact of disability and aims to inform the design of disability inclusive development programs. This paper reports the psychometric evaluation of the RAD.

**Methods:**

The initial version of the RAD comprised five sections: 1) demographics, 2) functioning, 3) rights awareness, 4) well-being, and 5) access to the community. Item functioning and construct validity were assessed in a population-based study in Bangladesh. Data were analysed using descriptive statistics (sections 2 and 5) and Rasch modelling (sections 3 and 4). A subsequent case–control study in Fiji tested the refined questionnaire in a cross-cultural setting and assessed the sensitivity and specificity of the RAD section 2 to identify people with disability.

**Results:**

2,057 adults took part in the study (1,855 in Bangladesh and 202 in Fiji). The prevalence of disability estimated using RAD section 2 in Bangladesh was 10.5% (95% CI 8.8-12.2), with satisfactory sensitivity and specificity (62.4% and 81.2%, respectively). Section 3 exhibited multidimensionality and poor differentiation between levels of rights awareness in both Bangladesh (person separation index [PSI] = 0.71) and Fiji (PSI = 0.0), and was unable to distinguish between people with and without disability (Bangladesh p = 0.786, Fiji p = 0.403). This section was subsequently removed from the questionnaire pending re-development. Section 4 had good ability to differentiate between levels of well-being (PSI = 0.82). In both countries, people with disability had significantly worse well-being scores than people without disability (p < 0.001) and also access to all sectors of community except legal assistance, drinking water and toilets (p < 0.001).

**Conclusions:**

Filed-testing in Bangladesh and Fiji confirmed the psychometric robustness of functioning, well-being, and community access sections of the RAD. Information from the questionnaire can be used to inform and evaluate disability inclusive development programs.

## Background

The World report on disability estimates 15% of the world’s population is living with disability, the majority of whom live in developing countries [1]. The prevalence of people with disability is expected to increase with the growing ageing population, increasing prevalence of chronic health conditions such as diabetes and cardiovascular disorders, and other factors such as road traffic accidents, natural disasters and conflict [1]. People with disability in most parts of the world experience inequality and discrimination and are excluded from health, education and employment opportunities [1]. There has been an increasing focus on disability inclusive development within many developing countries and from development partners in line with Article 32 of the United Nations Convention on the Rights of Persons with Disabilities (UNCRPD) [2]. Yet people with disability have not been adequately included in development activities, despite increasing evidence on the relationship between poverty and disability [3].

Planning strategies for disability inclusive development requires reliable information on both the magnitude of disability and the barriers which limit access to the community. Appropriate tools are also needed to enable measurement of changes resulting from disability-inclusive development. Currently there are limited comparable data on the prevalence and trends of disability across and within countries because of variations in definitions of disability and the methods used to collect data [4]. The International Classification of Functioning, Disability and Health (ICF) provides a common language and conceptualises disability as an outcome of the interaction between the health condition and contextual factors. Recognising the need for comparable measures of disability, the Washington Group on Disability Statistics (WG) developed a short set of disability measures for use with adults in national censuses and surveys and an extended set of questions which can be used to provide supplementary information [5]. The short set comprises six items related to limitations in the most basic actions or functions: seeing, hearing, walking, remembering, self-care, and communication. While data from that questionnaire can be used to estimate the prevalence of functional limitation in basic activities, it does not provide information on other dimensions of disability such as participation and the contextual factors that influence participation [6]. Such information could either be obtained by further analysis of functional difficulties with other measures of participation such as health, employment and education status among people with and without disability from censuses [5]. Other methods to obtain such data are by conducting extensive surveys such as the National Disability Survey in Afghanistan (NDSA) [7]. The NDSA survey was developed based on the ICF model [8] and Sen’s proposed capability approach [9] and it includes several modules. The costs and time required to implement such extensive surveys prohibit their use for smaller-scale development programs seeking to understand the situation of people with disability and to measure the impact of their programs on the lives of people with disability.

The Rapid Assessment of Disability (RAD) questionnaire was developed to support the design, implementation and evaluation of disability inclusive development activities [10]. Disability-inclusive development aims to increase participation of people with disability in their communities, for example in livelihoods, health and education as well as other areas of life such as religion, justice and social activities [11]. To enable this, programs need to be able to identify people with disability and understand their level of functioning, participation and access to services. Such information enables programs to not only plan in order to target the needs of people with disability but also address the barriers which restrict access to services and participation.

Goujon et al. [10] and Huq et al. [12] have described the development of the RAD questionnaire. This paper reports the field-testing of the RAD questionnaire among adults (≥18 years old) in Bangladesh and Fiji.

## Methods

### Ethics approval

Ethics approval was obtained from the University of Melbourne Human Research Ethics Committee (Australia), the Royal Victorian Eye and Ear Hospital Human Research and Ethics Committee (Australia), the International Centre for Diarrhoeal Disease Research, Bangladesh (icddr, b) Ethical Review Committee (Bangladesh), and the Fijian National Research Ethics Review Committee (Fiji). The study was conducted in accordance with the tenets of the Declaration of Helsinki. All participants provided a written or verbal informed consent. In case of participants who were not literate or were unable to provide written consent their verbal consent was obtained, i.e. the consent form was read to participants and their verbal agreement was recorded by the interviewer in front of a witness.

### The RAD questionnaire

The RAD questionnaire was developed based on a literature review, expert panel workshops and in-depth interviews with people with disability [10, 12]. The RAD questionnaire is interviewer administered and is comprised of two parts: a household questionnaire administered to the head of the household assessing socio-economic indicators such as source of water, electricity, sanitation, housing materials and assets including durable goods (e.g. television, radio, bicycle), and ownership of house, land and cattle and an individual questionnaire administered to each of the eligible members of the household.

The initial version of the individual questionnaire comprised five sections 1) demographics, 2) self-assessment of functioning, 3) awareness of the rights of people with disability, 4) well-being, and 5) access to the community [12]. Section 1 included 25 items related to demographics such as age, gender, ethnicity, religion, marital status, education, occupation, health conditions and assistive devices used. Section 2 comprised 18 items related to functioning in eight domains: vision (1 item), hearing (1 item), communication (1 item), mobility (2 items), self-care (1 item), gross and fine motor (1 item), cognitive (4 items), appearance (1 item) and psychological distress (6 items). The items related to psychological distress are from the Kessler-6 scale [12, 13]. Each item asked the participants to report whether they experienced difficulty in functioning in the last six months even when using assistive devices available to them (e.g. difficulty seeing even if wearing glasses). Those who answered ‘yes’ to an item were then asked to rate the frequency of difficulty as ‘some of the time’, ‘most of the time’, or ‘all of the time’. Those who self-reported having difficulty ‘most’ or ‘all of the time’ to at least one item from the first seven domains or at least two items from the psychological distress domain were considered as people potentially experiencing disability. The cut off criteria was determined based on the recommendations from the WG for short set questions [14] and also based on the consensus among the research group.

The purpose of section 3 was to obtain information about the awareness of the rights of people with disability. Section 3 comprised 17 items related to awareness of the rights of people with disability such as the right to ‘access information’, ‘live in a safe home environment’, ‘go to school or study’, ‘access health care’, ‘marry’ and ‘have children’. Each question was phrased “To what extent do you have rights to ….?”, and the response scale was rated on ‘none’, ‘some’ and ‘all’ categories.

**Figure 1:**
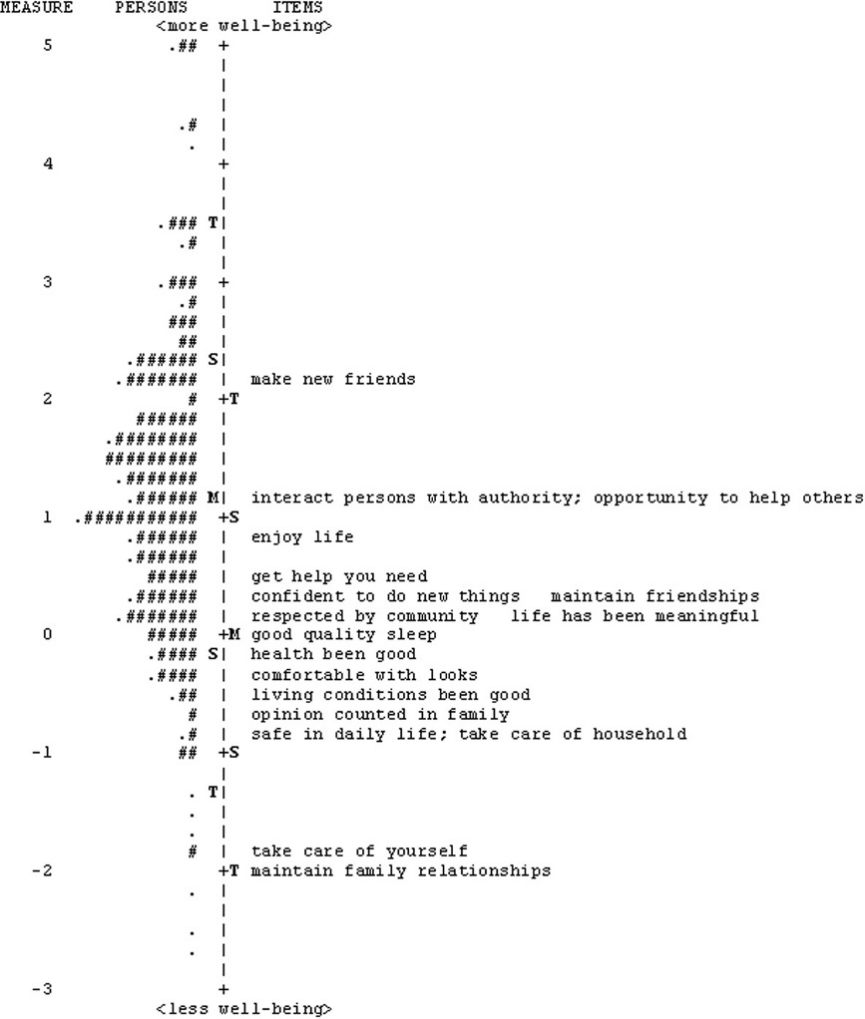
**Person-item map for the RAD section 4 (well-being) for Bangladesh sample.** To the left of the dashed line are the persons, represented by “#” and “.”, and on the right are the items. Each “#” represents 3 persons and each “.” represents 1 to 2 persons. Participants with higher well-being scores and items targeting higher levels of well-being are near the top of the diagram, while individuals with lower well-being scores and items targeting lower levels of well-being are near the bottom. M = mean; S = 1 standard deviation from the mean; T = 2 standard deviations from the mean.

Section 4 comprised 18 items to assess individuals’ perception of their well-being (Figure 1). The items included ‘good health’, ‘making friends’, ‘being safe in daily life’, ‘opinion counted in family’ and ‘taking care of one’s self’, ‘taking care of household’, ‘making new friends’, ‘maintaining family relationships’, and ‘respected in the community’ with the frequency of experiencing each situation reported on a four-category response scale ranging from ‘never’ to ‘all of the time’.

Section 5 asked respondents whether they had access to 14 community related domains such as access to health services, education and vocational training, work opportunities, social, legal, religious and rehabilitation. Each domain had three questions and the first question was phrased “In the last 6 months, did you have access to…… as much as you needed?” If they responded that they had not accessed services in this domain as much as they needed, they were asked to report reasons/barriers for this. Respondents were then asked which of the reasons given had limited access to the domain the most.

### Psychometric evaluation of the RAD questionnaire

The psychometric evaluation of the RAD questionnaire was conducted in two phases. The first phase involved field-testing in Bangladesh to assess the psychometric properties of the questionnaire. The findings of the Bangladesh field-testing study were used to identify poorly performing items, with the goal of removing or revising such items to enhance the psychometric robustness of the RAD. The second phase was conducted in Fiji to assess measurement properties of the revised questionnaire, to assess the sensitivity and specificity of the RAD to identify people with disability, and to assess the performance of the RAD in a cross-cultural setting. The findings from this phase of the study further informed modifications to the questionnaire and the development of the final version of the RAD.

#### Field-testing in Bangladesh

Field-testing in Bangladesh involved a cross-sectional population-based survey using two-stage cluster random sampling. The methodology was adapted from the Rapid Assessment of Avoidable Blindness (RAAB) survey, which was developed at the International Centre for Eye Health, London, as a rapid survey methodology to assess the prevalence and causes of vision impairment and cataract surgical coverage in a specific geographic area [15]. The first stage of sampling for the RAD field-testing involved randomly selecting clusters (villages or mahallas) from the sampling frame, with probability of selection proportional to cluster size. The sampling frame comprised all villages (in rural areas) and mahallas (in urban areas) in the Bogra district of Bangladesh, using population data from the 2001 national census projected to 2010. The second stage involved selecting households within clusters through compact segment sampling. Each village and mahalla was divided into equal segments through mapping of the sites so that each segment comprised approximately 50 people. Segments to be included in the study were selected by drawing lots. All households in the segment were included in the sample sequentially, with all eligible people in a household invited to participate in the study. If fewer than 50 eligible and consenting individuals were identified in a given segment, sampling continued in the next nearest segment until 50 people were recruited in a cluster. Eligibility criteria included people aged 18 years and above who had been living in the selected household for at least 3 months of the year. At least two return visits were made to recruit absentees.

In participating households, the household questionnaire was first administered to the head of each household. All eligible members of the household, including the head of the family, were then administered sections 1 and 2 of the individual questionnaire. Individuals identified as having disability in section 2 were then administered sections 3, 4 and 5. For each participant identified as having disability, an age and gender matched control was recruited from the neighbouring household that did not house a person with disability and all sections were administered. This was to allow the extent to which people with disability experience same opportunities when compared to their age and gender matched controls.

For field testing in Bangladesh, the questionnaire was translated into Bangla and then back translated into English.

#### Field-testing in Fiji

Considering that there are no gold standard instruments against which to compare the RAD questionnaire, a convenience sample of known people with disability were recruited in Fiji to test the sensitivity and specificity of the proposed cut off criteria to identify people with disability. The Fiji National Disability Policy [16] defines people with disability “are persons with long term physical, mental, learning, intellectual and sensory impairments and whose participation in everyday life as well as enjoyment of human rights are limited, due to socio-economic, environmental and attitudinal barriers”. Members of registered Disabled People’s Organisations self-identifying as people living with significant impairment were recruited as cases. Participants recruited as cases were people with a range of impairments who were receiving rehabilitation services from community-based rehabilitation programs. Controls were recruited through formal and informal networks of participating organisations. The two groups were individually matched on age and gender.

Prior to the study, the modified (following Bangladesh field-testing) version of the RAD individual questionnaire was reviewed by an advisory panel in Fiji to check for its cultural relevance and appropriateness of the items and language. Apart from the questions on section 1 (e.g. ethnicity, religion) no other changes were made. All sections of the individual questionnaire were administered to all participants.

### Training of interviewers

Field supervisors and interviewers were recruited based on their skills and previous experience. Some of the interviewers were people with disability. Training was provided for a week that included disability inclusion, study design, recruitment of participants, administration of the RAD questionnaire, ethics in research and collecting survey data, conducting interviews, data storage and referral mechanisms for participants. Supervised field practice sessions were also conducted as part of the training.

### Statistical analysis

Household questionnaire data from the Bangladesh study were used to derive a household wealth index using principal component analysis (PCA). Individuals were then classified into one of the three socio-economic groups based on values of the wealth index: the bottom 40% as poor, the next 40% as middle and the top 20% as rich [17, 18]. Descriptive statistical analysis was performed on the socio-demographic variables in section 1. Paired samples t-tests (for continuous variables) and generalised estimating equations (for categorical variables) were used to compare socio-demographic characteristics of people with disability and their matched controls.

Locations of category thresholds [19] for items from section 2 were analysed to examine whether participants could discriminate between response categories. Following this evaluation, responses were converted to binary variables of having a disability (difficulty ‘most’ or ‘all of the time’) or not (no difficulty or ‘some of the time’). Inter-item correlations (point biserial correlation coefficients for dichotomous variables) between section 2 items were examined to identify redundant items. Correlations of 0.75 or higher were interpreted as indicating redundancy.

Sensitivity and specificity of the cut off criteria used in RAD (i.e. self-reported difficulty ‘most’ or ‘all of the time’ to at least one item from the physical/sensory/cognitive domains or at least two items from the psychological distress domain of section 2) was assessed using data from Fijian field study. Sensitivity was calculated as the proportion of participants with disability identified by the RAD questionnaire among cases. Specificity was defined as the proportion of participants not identified to have disability by the RAD questionnaire among controls. Given that the RAD is intended for use as a population-level screening tool, we considered sensitivity and specificity values 60%-69% to be acceptable, 70%-79% good, 80%-89% very good, and ≥90% excellent.

Rasch modelling, a form of item response theory (IRT) that transforms ordinal scores into interval-level estimates [19, 20] (in logit units), was used to test construct validity of the sections 3 and 4 through the assessment of threshold ordering, unidimensionality, and targeting. Only sections 3 and 4 were subjected to Rasch analysis as these are the only sections of the RAD intended to be used for calculating section totals by summing responses across section items to capture participants’ perceived access to rights and well-being, respectively.

The Andrich rating scale model was used to obtain estimates of the level of characteristic (i.e., awareness of rights and well-being) represented by each item (item measure) and perceived level of these characteristics for each participant (person measure). Locations of category thresholds [19] were also estimated and items within each section were assessed for the evidence of disordered thresholds and to examine whether participants could discriminate between response categories. The response categories at least 1.4 logits apart were considered to be clearly differentiated by participants [21].

Measurement precision of the two sections, i.e. the ability to distinguish different levels of underlying characteristic, was assessed by the person separation index (PSI) and person separation reliability (PSR) scores. A PSI of ≥2.0 and a PSR ≥0.8 are considered minimally acceptable levels of separation, indicating that the questionnaire can distinguish at least three different levels of the characteristic of interest [20].

The extent to which each section measured a single underlying concept (i.e., section unidimensionality) was evaluated using item fit statistics and PCA of residual correlations. Item fit was assessed with the information-weighted (infit) mean-square (MnSq) statistics and standardised fit residuals (Zstd), both of which measure the discrepancy between the observed responses and responses predicted from the Rasch model. MnSq values of 0.7 to 1.3 and Zstd values of −2.0 to 2.0 were considered acceptable [22]. Values below the lower limit indicate redundancy and values above the upper limit indicate unacceptable level of variability in the responses (high measurement error). When a scale functions as intended, with all items measuring a single dimension, this single dimension should explain most of the correlations in the item set. The existence of any additional dimensions is inferred through the assessment of residual variance (variance unexplained by the primary dimension) using PCA. Unidimensionality was inferred if 1) the primary dimension explained at least 50% of the variance in the responses to the section items and 2) the first residual component had an eigenvalue <2.0, indicating that variance explained by this residual component carries less information than 2 items, the smallest number of items needed to form a dimension [23].

The capacity of the items to adequately represent the full continuum of a characteristic of interest is called targeting and was assessed by visual inspection of the person-item map and the difference between means of person and item measures. For a perfectly targeting instrument, the difference in means would be 0; an absolute difference of >1.0 logits indicates significant mistargeting and occurs when the amount of characteristics in the sample is substantially higher or lower than the average level of the same characteristic targeted by the items [24].

Sections 3, 4 and 5 were assessed on known-groups validity by comparing the scores of people with disability and controls using paired-samples t-tests (sections 3 and 4) and generalised estimating equations (section 5). It was expected that people with disability would score significantly higher on each section than their matched controls.

Rasch analysis was performed using Winsteps version 3.74 (Winsteps, Chicago, IL). Remaining statistical analyses were performed using PASW Statistics 18 (PASW Statistics for Windows, SPSS Inc., Chicago, IL).

## Results

### Questionnaire administration

Field testing of the RAD sampling and interview methodology showed that, on average, the full interview (sections 1–4) takes approximately 45 minutes to complete, while the basic version (sections 1–2) takes approximately 20 minutes. Thus, a survey team comprising three interviewers is able to interview 50 people (one segment) in a day.

### Participants

Participant demographics in the Bangladesh and Fiji are presented in Table 1. A total of 1,855 adult participants (80% response rate) were recruited from 66 clusters in Bangladesh. Participants’ mean (±SD) age was 38.6 (±16.2) years (Table 1). Of these, 195 (10.5%, 95% CI: 8.8, 12.2) individuals were identified to have disability based on their responses to the RAD section 2. Frequency of self-reported functional limitations ranged from 11 (0.6%) on communication difficulties to 103 (5.5%) on seeing difficulties. The numbers of participants who reported difficulties on physical/sensory/cognitive domains and psychological distress domain were 190 (10.2%) and 101 (5.4%) respectively. There was no significant difference between people with disability and their matched controls (n = 195) in the level of education but people with disability were more likely to be unemployed (26.6% vs 8.9%, p < 0.001).

**Table 1:** **Participant demographics in Bangladesh and Fiji**

Socio-demographics		Bangladesh				Fiji		
		Total sample N = 1855 n (%)	Cases N = 195 n (%)	Controls N = 195 n (%)	P*	Cases N = 101 n (%)	Controls N = 101 n (%)	P*
Age (mean ± SD) years		38.6 ± 16.2	50.0 ± 17.19	51.5 ± 18.5	0.411	44.2 ± 15.3	44.1 ± 15.8	0.763
Gender	Male	763 (41.1)	83 (42.6)	83 (42.6)	1.000	54 (53.5)	45 (44.6)	0.354
	Female	1092 (58.9)	112 (57.4)	112 (57.4)		47 (46.5)	53 (52.5)	
Education	None	679 (36.6)	99 (50.8)	92 (47.2)	0.898	19 (18.8)	3 (3.0)	0.004
	1-4 years	372 (20.1)	42 (21.5)	43 (22.1)		12 (11.9)	7 (6.9)	
	5-9 years	588 (31.7)	40 (20.5)	44 (22.6)		27 (26.7)	26 (25.7)	
	≥10 years	216 (11.6)	14 (7.2)	16 (8.2)		29 (28.7)	48 (47.5)	
Occupation	Unemployed	128 (6.9)	51 (26.6)	17 (8.9)	<0.001	34 (33.7)	13 (12.9)	<0.001
	Farmer	343 (18.5)	26 (13.5)	46 (24.1)		12 (11.9)	9 (8.9)	
	Labourer	231 (12.5)	22 (11.5)	24 (12.6)		7 (6.9)	16 (15.8)	
	Homemaker	966 (52.1)	84 (43.8)	97 (50.8)		24 (23.8)	26 (25.7)	
	Other	113 (6.1)	9 (4.7)	7 (3.7)		20 (19.8)	36 (35.6)	
Socio-economic status	Rich	367 (19.8)	30 (15.4)	29 (14.9)	0.803	-	-	
	Middle	740 (39.9)	56 (28.7)	62 (31.8)		-	-	
	Poor	747 (40.3)	109 (55.9)	104 (53.3)		-	-	

*From paired sample t test (continuous variables) or generalised estimating equations models (categorical variables), comparing people with disability and their matched controls.

A total of 202 adults were recruited in Fiji and of these, 101 were cases (mean ± SD age: 44.2 ± 15.3 years) and 101 were controls (mean ± SD age: 44.1 ± 15.8 years) (Table 1). The numbers of participants who reported difficulties on physical/sensory/cognitive domains and psychological distress domain were 60 (59.4%) and 27 (26.7%) respectively. Cases had significantly lower education levels (no education: 18.8% vs 3.0%, 10 years or more: 28.7% vs 47.5%, p = 0.004) and significantly higher frequency of unemployment (33.7% vs 12.9%, p < 0.001) compared to controls.

### Section 2: self-assessment of functioning

**Figure 2:**
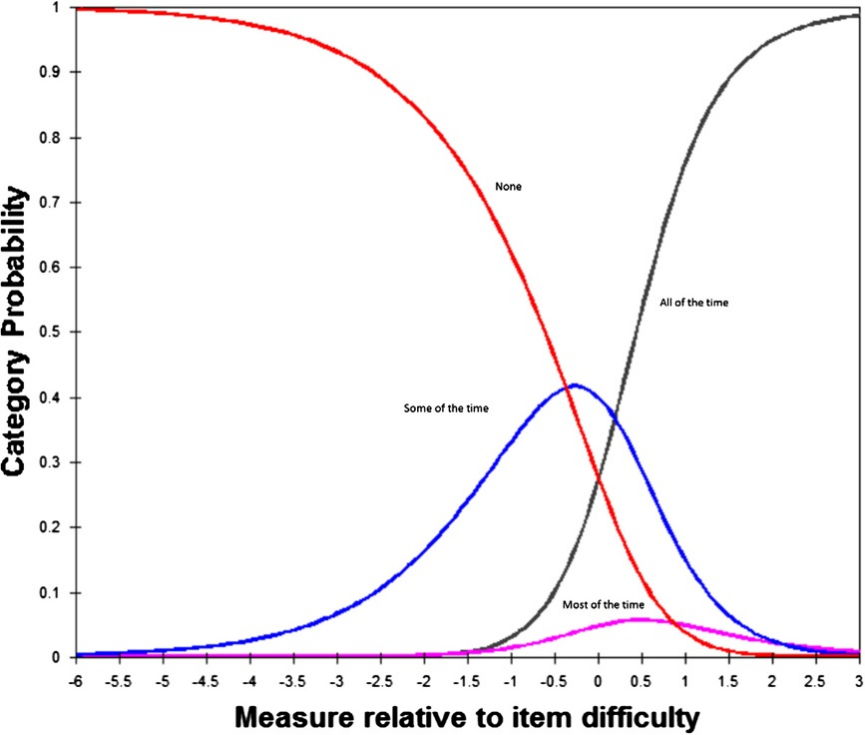
**Category probability curves showing disordered thresholds for four response categories of frequency of difficulty ratings in section 2.**

The average locations of response categories for the 18 items in section 2 along the disability continuum were analysed (Figure 2). There was substantial separation in the average locations of the two lowest response categories (0 = never and 1 = some of the time) and between the second and third (2 = most of the time and 3 = all of the time) response categories. Response categories 2 and 3 appeared to be very close to each other on the disability continuum, indicating that these response categories could not be distinguished by the respondents and were representing similar level of disability.

#### Inter-item correlations

Inter-item correlations were found to be high between the items ‘moving around inside home’ and ‘moving around outside’ (r = 0.92, p < 0.001) indicating redundancy between the items. Additionally, ‘self-care’ item had strong inter-item correlations (ranging from r = 0.50 to 0.91, p < 0.001) with physical and cognitive functioning items. Following consultations with disability experts, it was decided to remove ‘moving around inside home’ and ‘self-care’ items from the questionnaire. Removal of these items from section 2 produced virtually no change in the estimate of prevalence of disability (10.4%, 95% CI: 8.7, 12.0). Therefore, these two items were omitted from the RAD prior to field testing in Fiji.

#### Sensitivity and specificity

Using the RAD cut off criteria for disability, 63 (62.4%) participants recruited as cases were identified to have disability (true positives, sensitivity) and 82 (81.2%) controls were identified as not having disability (true negatives, specificity) in Fiji, indicating acceptable sensitivity and very good specificity in detecting disability. Twenty three out of 38 (60.5%) people who were identified as false negatives reported mild functional limitations related to mobility and some were using assistive devices such as crutches. None of these participants reported difficulties on the well-being or access sections.

Of the 19 (18.8%) participants who were identified to have disability among the controls (false positives), six (31.6%) were aged >65 years and reported difficulties related to sensory, cognitive and physical aspects of functioning; two (10.5%) participants reported difficulty seeing, of which one needed a new glasses prescription; three (15.7%) participants had also reported bothering health conditions such as heart disease, diabetes, and high blood pressure and reported high levels psychological distress on section 2 items; and eight (42.1%) reported difficulties with cognitive aspects and psychological distress. These participants also reported difficulties on the well-being or access sections.

### Section 3: awareness of the rights of people with disability

Examination of the average locations of response categories of the 17 items in section 3 on the awareness of rights continuum showed that while there was no threshold disordering, the average locations of the two lowest response categories (1 = none and 2 = some) were very close to each other (<1.4 logits separation). This indicated that participants could not discriminate three response categories, but used them as a dichotomous scale (yes/no). Category 1 (none) had large amount of random error (MnSq 1.34) with only 7% of the sample endorsing this response option. The most frequently endorsed response category was category 3 (all), which was endorsed by 48.8% to 82.9% of respondents depending on the item, indicating strong ceiling effects and/or bias towards acquiescent responding. The mean (±SD) person measure was 1.99 (±1.11) in people with disability and 2.03 (±0.10) logits in controls and there was no statistically significant difference between the two groups (p = 0.786).

The precision of the scale for measuring levels of rights awareness was poor, with PSI (1.58) and PSR (0.71) lower than the acceptable range (≥2.0 for PSI and ≥0.8 for PSR), indicating that the item set had low ability to discriminate between people with high and low levels of awareness of their rights. Section 3 also exhibited evidence of multidimensionality. The item ‘rights to have children if you want to’ had high amount of random error (MnSq 1.20, Zstd 3.20) and items ‘rights to make decisions about your own life’ (MnSq 0.82, Zstd −2.30), ‘rights to your opinion counting in family decisions’ (MnSq 0.84, Zstd −2.10) and ‘rights to be treated the same way as everyone else in society’ (MnSq 0.83, Zstd −2.60) showed evidence of redundancy. On PCA of the residuals, total variance explained by the item set was 32.9% and the eigenvalue of the first residual component was 2.1 further suggesting multidimensionality of the items set. However, when the Rasch scores of this section were further explored using exploratory factor analysis no meaningful separation of items into dimensions had emerged.

#### Revision and field testing in Fiji

Given the suboptimal psychometric properties of section 3 in the Bangladesh field study and the tendency to elicit responses towards high levels of rights awareness in both controls and people with disability, the phrasing of the questions in this sections was changed to “Do people with disability have the right to …?” prior to field testing in Fiji. Additionally, the categories were dichotomised to ‘yes’ or ‘no’ because there was no significant separation between thresholds of categories ‘none’ and ‘some’ in the previous version.

Despite the changes, the majority (ranging from 92.8% to 98.1%) of the respondents in Fiji had responded positively to the questions of this section of the RAD. The respondent’s preference for affirmative responses has resulted in very poor person separation (PSI = 0 and PSR = 0) indicating that the scale could not discriminate between different levels (yes/no) of rights awareness. On PCA of the residuals, total variance explained by the item set was 36.3% and the eigenvalue of the first residual component was 3.1, further suggesting multidimensionality in this section, similar to the Bangladesh study. The mean person measure was 3.85 (±0.16) logits in people with disability and 4.01 (±0.11) logits in controls and there was no statistically significant difference between the two groups (p = 0.403).

Following the results of field testing in Fiji, and the need for more research on how to accurately measure awareness of rights of people with disability, this section has been removed from the final version of the RAD.

### Section 4: well-being

The categories (1 = never, 2 = some of the time, 3 = most of the time and 4 = all of the time) were reversed for Rasch analysis so that higher values on the logit scale corresponded to higher levels of well-being. The response category thresholds were ordered as expected and the precision of the scale was good, with PSI 2.14 and PSR 0.82, supporting the ability of the scale to discriminate at least three levels of well-being. The most difficult item (corresponding to the highest level of well-being) was ‘able to make new friends’ (2.13 logits) and the least difficult item (the lowest level of well-being) was ‘maintaining family relationships’ (−1.95 logits). There were two misfitting items: ‘able to make new friends’ (MnSq 1.52, Zstd 7.1) and ‘able to maintain friendships’ (MnSq 1.39, Zstd 5.5). However, PCA of the residuals revealed that the variance explained by the items was 58% and the eigenvalue of the first residual component was 1.9 supporting unidimensionality of the scale. The ‘get the help you need’ item was found to be redundant with the ‘opportunity to help’ item (residual correlation of 0.3).

The mean ± SD person measure of 1.18 ± 1.34 indicated that the average level of perceived well-being of the sample was better than that represented by the items, which was unexpected given that people without disability comprised half of the sample. However, the targeting has been satisfactory with reasonably good spread of items along the well-being continuum of the sample (Figure 1) and people with disability had significantly poorer well-being scores compared to the controls (mean difference = 0.97, p < 0.001).

#### Revision and field testing in Fiji

Following consultations with disability experts and prior to field testing in Fiji, the ‘able to maintain friendships’ item was removed from the questionnaire. However, it was decided that ‘able to make new friends’ should be retained in section 4 to maintain good spread of the items across levels of well-being. The items ‘getting the help you need’ and ‘opportunity to help’ were also retained, to be further assessed in Fijian Study.

The 17-item version of section 4 tested in Fiji showed that the categories were ordered as expected, and were at least 1.4 logits apart, indicating good discrimination of the categories. The revised version of section 4 also had good measurement precision, with PSI 2.20 and PSR 0.83. Only one item, ‘get the help you need’, was misfitting with the rest of scale items, displaying random noise (MnSq 1.84, Zstd 6.25). PCA of the residuals revealed that the variance explained by the item set was slightly lower than optimal (45.5%) but the eigenvalue of the first residual component was only 1.9. The mean ± SD person measure was 1.53 ± 1.71, which was higher than the expected average possibly because 50% of the sample was comprised of people with disability who had rehabilitation interventions. However, similar to the results obtained for a Bangladesh sample, people with disability had poorer well-being scores compared to the controls (mean difference = 0.92, p < 0.001).

Following the results of field testing in Fiji, the ‘get the help you need’ item was removed from section 4, and the remaining 16 items were included in the final version of the RAD.

### Section 5: access to the community

Table 2 presents the responses for access to each sector for Bangladesh and Fiji samples. There was significantly worse access to the community for people with disability compared to controls on all sectors, except legal assistance, drinking water and toilets, in both Bangladesh and Fiji (p < 0.001). This section did not have any modifications following field-testings.

**Table 2:** **Access to the community for people with disability and controls in Bangladesh and Fiji**

Services		Bangladesh			Fiji		
		Cases n (%)	Control n (%)	P*	Cases n (%)	Control n (%)	P*
Work	Yes	72 (37.5)	93 (47.7)		67 (66.3)	87 (87.9)	
	No	29 (15.1)	4 (2.1)	<0.001	20 (19.8)	9 (9.1)	0.001
	Not needed	91 (47.4)	98 (50.3)		14 (13.9)	3 (3.0)	
Education	Yes	4 (2.1)	12 (6.2)		31 (30.7)	60 (61.2)	
	No	41 (21.4)	27 (13.8)	0.035	28 (28.3)	12 (12.2)	0.004
	Not needed	147 (76.6)	156 (80.0)		45 (44.6)	26 (26.5)	
Health services	Yes	132 (68.8)	129 (66.2)		83 (82.2)	80 (81.6)	
	No	28 (14.6)	5 (2.6)	<0.001	15 (14.9)	5 (5.1)	<0.001
	Not needed	32 (16.7)	61 (31.3)		3 (3.0)	13 (13.3)	
Participate in community consultations	Yes	93 (48.4)	119 (61.0)		52 (51.5)	82 (82.8)	
	No	38 (19.8)	9 (4.6)	<0.001	35 (34.7)	8 (8.1)	<0.001
	Not needed	67 (34.4)	61 (31.8)		14 (13.9)	9 (9.1)	
Assistive devices	Yes	23 (12.0)	13 (6.7)		35 (35.0)	19 (19.2)	
	No	70 (36.5)	12 (6.2)	<0.001	33 (33.0)	7 (7.1)	<0.001
	Not needed	99 (51.6)	170 (87.2)		32 (32.0)	73 (73.7)	
Rehabilitation	Yes	13 (6.8)	5 (2.6)		46 (45.5)	14 (14.1)	
	No	48 (25.0)	9 (4.6)	<0.001	27 (26.7)	11(11.1)	<0.001
	Not needed	131 (68.2)	181 (92.8)		28 (27.7)	74 (74.7)	
Safe/drinking water	Yes	192 (100)	193 (99.0)		97 (96.0)	97 (98.0)	0.421
	No	0 (0.0)	2 (1.0)	0.159	4 (4.0)	2 (2.0)	
Toilet facilities	Yes	179 (93.2)	189 (96.9)		93 (92.1)	96 (97.0)	0.129
	No	14 (6.8)	6 (3.1)	0.093	8 (7.9)	3 (3.0)	
Disaster management	Yes	10 (5.2)	7 (3.6)		36 (36.4)	64 (64.6)	
	No	36 (18.8)	13 (6.7)	0.001	45 (45.5)	15 (15.2)	<0.001
	Not needed	146 (76.0)	175 (89.7)		18 (18.2)	20 (20.2)	
Legal assistance	Yes	14 (7.3)	13 (6.7)		34 (34.3)	35 (35.4)	
	No	2 (1.0)	4 (2.1)	0.708	18 (18.2)	8 (8.1)	0.098
	Not needed	176 (91.7)	178 (91.3)		47 (47.5)	56 (56.6)	
Social activities	Yes	135 (70.3)	161 (82.6)		64 (64.0)	91 (91.9)	<0.001
	No	19 (9.9)	5 (2.6)	0.003	20 (20.0)	3 (3.0)	
	Not needed	38 (19.8)	29 (14.9)		16 (16.0)	5 (5.1)	
Religious activities	Yes	176 (91.7)	187 (95.9)		81 (80.2)	95 (96.0)	
	No	14 (7.3)	3 (1.5)	0.013	14 (13.9)	2 (2.0)	0.002
	Not needed	2 (1.0)	5 (2.6)		6 (5.9)	2 (2.0)	
Government social welfare	Yes	17 (8.9)	10 (5.1)		56 (56.4)	25 (25.3)	
	No	46 (24.0)	14 (7.2)	<0.001	29 (28.7)	10 (10.1)	<0.001
	Not needed	76 (39.6)	121 (62.1)		14 (3.9)	64 (64.6)	
	Do not know	53 (27.6)	50 (25.6)		2 (2.0)	0	
Disabled persons organisations	Yes	8 (4.2)	0 (0.0)		30 (30.3)	8 (8.3)	
	No	36 (18.8)	5 (2.6)	<0.001	35 (35.4)	12 (12.5)	<0.001
	Not Needed	75 (39.1)	119 (61.0)		6 (6.1)	44 (45.8)	
	Do not know	73 (38.0)	71 (36.4)		28 (28.3)	32 (33.3)	

*P-values are from generalised estimating equations modelling (logistic regression), comparing people with disability and their matched controls.

## Discussion

This study reports psychometric evaluation of the RAD questionnaire – a measure of disability that provides information on functional limitations and their impact on lives of people with disability – in Bangladeshi and Fijian cultures. The results largely support the construct validity and cross-cultural applicability of the RAD. The questionnaire has acceptable levels of accuracy in identifying people with disability, with sensitivity and specificity of cut off criteria 62.4% and 81.2%, respectively. Section 4 was evaluated using Rasch modelling, with results providing evidence of appropriate category function for response categories, unidimensionality of the item set, ability to distinguish between at least 3 levels of well-being, and ability to differentiate people with and without disability. Section 5 was also able to demonstrate construct validity, with significantly worse access to the community among people with disability compared to their matched controls in both cultures. An exception was section 3, which exhibited poor separation of response categories, multidimensionality, and lack of differentiation between levels of rights awareness and was unable to distinguish between people with and without disability. Considering the psychometric shortcomings, the rights awareness section has been removed from the final version of the RAD. The current version of the RAD questionnaire has four individual assessment sections: demographics, self-reported functional limitations, well-being, and access to the community.

In the Bangladesh phase of this study, 195 (10.5%, 95% CI: 8.8, 12.2) participants were found to have disability using RAD section 2 items. After adjusting for sample weights as determined based on the population census of 2010, the prevalence of disability is 8.91% (95% CI: 7.34, 10.58) in adults aged 18 years or older. This estimate is similar to the 9.1% among adults and children obtained in the 2010 Household Income and Expenditure Survey (HIES), using the WG short set questionnaire [25]. Similar to the HIES survey the RAD had also identified that seeing difficulties is the most common form of disability. While estimates of disability prevalence obtained from the RAD and WG concur, the RAD collects information on a broader range of functional limitations, containing items related to psychological distress in addition to physical, sensory and cognitive domains. With an estimated 5.4% prevalence of psychological distress using Kessler-6 items [13] in section 2, the ability of the RAD to identify psychological aspects of disability can provide useful additional information on the extent of different types of disability, and potentially allow tailoring disability inclusive development programs to meet needs linked to specific disabilities.

The Fiji study demonstrated that participants with a mild level of functional difficulties could be missed using the RAD cut off criteria. However, the majority of false negatives in section 2 (23/38, 60.5%) did not report difficulties in well-being and access to the community sections. On the contrary, false positives among the control group reported functional limitations that adversely impacted their well-being and community access. It could be possible that people with obvious functional limitations (e.g. physical conditions) are more likely to register with DPOs and less obvious functional limitations such as psychological distress or sensory limitations could be missed. This might have affected recruitment as cases and controls and this is one of the limitations of this study. While the RAD may be underestimating mild level of functional difficulties, it is in fact able to identify those who have significantly lower access to opportunities to participate in the community.

Data from the section 2 considers the person as the unit of analysis and establishes presence of disability based only on self-reported difficulty in functioning at the person level, and this is a limitation. However, the RAD provides the opportunity to assess other aspects of functioning and barriers to participation in the community. Such data will be useful at the program level. Level of functioning and access to services could be measured over time to investigate changes following an intervention. However, further research is needed to determine of the responsiveness of the tool to measure change.

Section 3 was developed with the intention that it will help disability inclusive development initiatives to assess awareness of rights of people with disability in the community. Items in this section were developed based on current policies and frameworks, expert panel workshops and qualitative interviews with people with disability [12]. However, results of this study identified a strong tendency towards acquiescent responding, regardless of the format or structure of the questions in this section. Since rights awareness is an important component of disability inclusive development, further research and studies are needed to re-develop section 3 of the RAD. Such studies could involve focus group discussions with people with disability to identify issues specifically related to violation of rights of people with disability and identify appropriate phrasing of the questions. Although people with disability were involved in the expert panel discussions and qualitative interviews during RAD development [12], they were active members of disability related organisations who are well aware of the rights of people with disability and the appropriate terminology. It may also be possible that awareness of rights cannot be assessed using closed ended questionnaires but may require in-depth interviews. In the interim, we have retained an open-ended question in section 2 that asks the respondent’s knowledge on the rights of people with disability. Information related to the current situation regarding access to enacted rights for people with disability can still be assessed by disability inclusive development programs using the information in the ‘access to the community’ section that has questions related to accessing services and participating in the social and community life [26].

Section 4 was found to be a psychometrically robust tool to measure well-being in cross-cultural settings. Using the scores in sections 4 and 5 in conjunction with demographics and section 2 would provide a comprehensive picture of the extent and impact of disability, capturing different dimensions of disability. Further studies are also needed to test the temporal stability of the RAD sections, their ability to detect changes when used before and after an intervention, and to corroborate the construct validity of the RAD questionnaire through the assessment of convergent and discriminant validity. Future studies would also need to test the sensitivity and specificity of section 2 to assess specific types of functional limitations, such as vision or hearing impairment.

The RAD questionnaire is intended to provide a snapshot of the life situation of people with disability and can be used as a tool for obtaining baseline data before designing and implementing disability inclusive development strategies. Unlike most disability measurement surveys that rely on asking screening questions to the head of the household, the RAD is administered to individuals within a household. Although proxy responses are less time-consuming at a household level they may risk underestimation of disability and its impact. Field testing of the RAD sampling and interview administration methodology in a population-based study in Bangladesh showed that two-stage cluster random sampling with compact segment design allows recruitment of 50 people per cluster segment in a day with a team of three interviewers. Only people who are identified as having a disability based on section 2 of the questionnaire are administered the complete questionnaire, with average administration time of 45 minutes. Using this methodology, we were able to complete the survey in Bangladesh in 6 weeks. While this supports the ability of the RAD for ‘rapid’ collection of disability-related data, the total time taken to complete a given study will depend on the size and geographic distribution of the area, population density, and available resources.

The results of this study show that the RAD questionnaire offers an efficient method to collect comprehensive information on the magnitude of disability, including access to different sectors in the community. However, it should be noted that the RAD does not collect information on the causes of disability, the reasons for participation restrictions or the need for specific services. If such information is of interest, targeted specific questions will be needed to obtain further information. Although the RAD questionnaire has been tested in two countries, cultural adaptation and cognitive testing of the questionnaire are recommended when RAD will be used in other countries.

## Conclusions

The RAD questionnaire was developed to identify people with disability, and for gathering information about their level of participation specifically related to well-being and access to the community in comparison with those without disability in the community. Field testing in Bangladesh and Fiji found the self-assessment of functioning and well-being sections to be psychometrically robust. Access to the community section was useful to identify the differences in access to different services and participation in the community between people with and without disability. However, questions developed to assess the awareness of rights were found to not yield usable data at this stage.

Information from the RAD can be used to design disability inclusive development programs, and to provide baseline data against which to measure the programs’ impact. Using the RAD in the context of development programs will allow further assessment of its utility as a tool for informing and capturing effectiveness of disability inclusive development programs.

## Abbreviations

icddr: b: International Centre for Diarrhoeal Disease Research, Bangladesh
ICF: International Classification of Functioning, Disability and Health
MnSq: Mean Square Statistics
NDSA: National Disability Survey in Afghanistan
PCA: Principal Component Analysis
PSI: Person Separation Index
PSR: Person Separation Reliability
RAD: Rapid Assessment of Disability
UNCRPD: United Nations Convention on the Rights of Persons with Disabilities
WG: Washington Group on Disability Statistics.
